# Social sustainability in Bangladesh marine fisheries management: A case from Hatiya fishing community

**DOI:** 10.1016/j.heliyon.2024.e34124

**Published:** 2024-07-04

**Authors:** Md Monirul Islam, Makidul Islam Khan, Gouri Mondal, Most Nilufa Yeasmin, Aparna Barman

**Affiliations:** a Department of Fisheries, University of Dhaka, Dhaka, 1000, Bangladesh; b Department of Fisheries and Marine Bioscience, Bangabandhu Sheikh Mujibur Rahman Science and Technology University, Gopalganj, 8100, Bangladesh

**Keywords:** Social sustainability, Marine protected area, Hatiya, Fisheries management, SDGs, Livelihood

## Abstract

Social sustainability in fisheries focuses on retaining or improving societal welfare in the fishery system without threatening its long-term financial benefits and socio-cultural welfare. However, often social sustainability issues are ignored while implementing any fisheries management actions rather than only focusing on economic and environmental sustainability issues. This study assesses the social sustainability in Bangladesh marine fisheries management gaining insights from the coastal and marine fisheries-dependent communities of Hatiya Upazila in 2021–2022 using a mixed method approach especially focusing on the social issues during the marine fishing ban. Results have shown positive effects on fish production but negative implications on the socio-economic circumstances of the reliant households after the implementation of the marine fishing ban. During the ban, the ability of around 33 % of fisher households to have 3 meals a day has drastically reduced to 2 or 1 meal per day. Households' average fish intake has reduced from 7 kg to 4 kg per week during the ban. Similarly, there have been detrimental effects on family relationships, healthcare access and children's education during the ban. Moreover, pregnant women and children have suffered greatly from protein deficiencies as fishers could not buy protein-rich foods for their families during the ban. Due to the shortage of alternate income-generating activities (AIGAs), almost 71 % of fishers became indebted during the ban. To assist the fishers during the ban, the government has implemented several measures, such as offering incentives (40 kg of rice per registered fisherman per month) and/or AIGA but those are too scant to recompense for the loss that is incurred due to the fishing ban. Finally, this study provides some way forward to bring social sustainability that is affected due to the marine fishing ban as well as to partly meet the targets of Sustainable Development Goals 1, 2, 14 and 15.

## 1 Introduction

Social sustainability is a more elusive and complex concept than environmental or economic sustainability. However, social sustainability lacks a precise, logical and applicable definition [1]. Various authors have attempted to define ‘social sustainability’. Polèse and Stren (2000) defined social sustainability as: “Development (and/or growth) that is compatible with the harmonious evolution of civil society, fostering an environment conducive to the compatible cohabitation of culturally and socially diverse groups while at the same time encouraging social integration, with improvements in the quality of life for all segments of the population” [2]. According to Vancouver's Social Development Practice, meeting the basic needs are prerequisite for a community to perform well and become sustainable and the communities must build and maintain their particular assets that have the resiliency to avert and/or address the forthcoming difficulties to be socially sustainable [3].

According to Vallance et al. (2011), we should comprehend the social sustainability processes and structures as existing and requiring further development using the following three approaches: (1) ‘development sustainability’ which discourses basic needs, the creation of social capital, justice, equity and so on; (2) ‘bridge sustainability’, guarantees that structures are updated to meet changing demands; and (3) ‘maintenance sustainability’ guarantees the preservation of functional and operating structures [4]. According to some researchers, social sustainability is an ethical code of conduct for human existence and development that must be met in a way that is inclusive, linked, equitable, and responsible [5]. Some scholars establish a link between social sustainability and the management of social resources, such as interpersonal competencies, interpersonal connections, and social values [6]. Sachs (1999) listed a number of components including equitable income, social homogeneity, gender equality, access to employment and products and services, etc. [7].

There are several suggested components of social sustainability, for example, equity, gender equality, social inclusion and interaction, safety and security, adaptability, etc. [3,[8], [9], [10]]. Among them, equity is seen as an essential component of social sustainability. The two aspects of equity—intergenerational and intragenerational equity—are highlighted in the literature on sustainability [8]. For social sustainability, both aspects are important. The term “intergenerational equity” describes the equitable sharing of resources to the current and future generations while intragenerational equity states the fairness in allocating assets among the competing interests at present. Besides, gender equality is another component of social sustainability. Ensuring gender equality amongst participants requires stepping up efforts to recruit and encourage more women [9]. One key strategy for promoting gender equity is the provision of full- and part-time jobs for men and women. Besides, good governance plays a significant role in bringing social sustainability. One crucial need for governability is social justice. In the absence of justice, stakeholders would probably rebel against government initiatives to protect the resources or advance development [11].

Like other communities, building and maintaining social sustainability in the fisheries communities is indispensable as vulnerable groups are prevalent in the fisheries sector. However, when executing any fisheries management-related activities, the social aspect of sustainability is occasionally neglected [[12], [13], [14]] or attained the least concern in natural resource management particularly in coastal and marine systems [[15], [16], [17]]. The environmental economic facets of sustainability have often dominated the discussion, while social dimensions have been marginalized because of two main reasons: 1) there is not enough information available to provide comprehensive images of the social components of sustainability for population across time, and 2) there is a failure to appreciate the relevance of social issues in sustainability [18]. Even though social sustainability is recognized for its social aspects—intergenerational equity, the structure of economic activity, and the entire rationale for economic development are at the heart of social phenomena—social sustainability assessments have not yet been applied to many fisheries worldwide, and in those that have, the process of creating the assessment instruments has often been sluggish [18]. Therefore, sustainability is typically presented as resting on three vital pillars: ecological, economic, and social, especially since the 1990s when John Elkington coined the expression “triple bottom line” [19]. Since its inception in 1991, the United Nations Code of Conduct for Responsible Fisheries has consistently taken social factors into account, in order to establish principles “for responsible fishing and fisheries activities, considering all related economic, biological, social, technological, environmental, and commercial aspects.” However, it has taken many years to establish and is only now accessible in recent years the guidelines for integrating social issues into fisheries management techniques to comply with the Code [18].

Bangladesh's government has established different acts, rules, ordinances, regulations and policies for the proper management of the fisheries sector [20]. Some specific approaches/strategies for managing the sector are the creation of marine protected areas (MPA), fish sanctuaries [21], co-management [22], community-based management [22], ecosystem approach to fisheries management (EAFM) [23], payment for ecosystem services, integrated coastal zone management [24], etc. The conservation activities comprise particular gear limits, bans on harvesting fish that are undersized, limits on when and where fishing is allowed and so on. The fishery-dependent communities are impacted by these conservation efforts in many ways [[25], [26], [27]]. For example, fishing restrictions have significant short-term negative impacts, especially on the income and way of life of vulnerable coastal fishermen and their communities [13,14,28,29], since there are no substitute jobs available while fishing is prohibited.

Since 2015, the Bangladesh government has imposed 65-days fishing ban in coastal and marine waters to protect marine fisheries, especially to increase the output of hilsa fisheries [30] as hilsa is the single largest fishery generates more than 1 % of the national GDP (Gross domestic product) and supports the livelihoods of over 2.5 million people, both directly and indirectly [31]. The spatio-temporal fishing restriction for 65 days in the coastal and marine waters has been proven to enhance or boost fisheries production as documented by the 15 or 22 days fishing prohibition to safeguard hilsa spawning [32,33]. Furthermore, the 65-day fishing ban considerably improves ecological conditions that particularly provide a favorable environment for boosting fish and shellfish production [34]. Alike Bangladesh, a similar fishing ban (60-day) in India has aided in the restoration of habitat and rebuilding of stock through recruitment [14]. However, the social consequences of the conservation program have gotten less attention, which has led to poorer performance in terms of social equity and efficacy [35,36]. In the projects related to fisheries and aquaculture management and development in Bangladesh, mostly the increase of fish production, quality control, conservation, disease management and overall macro-economic development are focused and social sustainability issues are often ignored or get less priority. As a result, concerns are frequently raised regarding environmental conservation and sustainability of fishers' livelihoods – battle or balance? However, scarce studies are available on the social impact of the 65-day marine fishing ban on the fishery-dependent communities of coastal Bangladesh such as Islam et al. (2023) [13]. As such, the overall objective of this study is to assess the impacts of the marine fishing ban on the social aspects of sustainability of the fishery-reliant peoples in Bangladesh and suggest ways for improvement.

## 2 Social sustainability in fisheries

A global census addressed that fisheries are facing environmental and socio-economic crises in many parts of the world [37]. The sustainable fishery is a potential way to face this crisis [38]. Though effective fisheries management can sustain the long-term viability of fisheries and the economic advantages they yield, emphasizing ecological sustainability alone runs the risk of ignoring the ultimate well-being goals of the world's fishers that require comprehensive social sustainability [29,39,40]. Social sustainability focuses on sustaining or improving societal welfare in the fishery system where the economic and socio-cultural well-being along with its cohesion and the long-term advantages related to human welfare are not compromised [41]. The primary concerns for a fisherman and his or her family are the security of income and jobs as well as the safeguarding of the right to fish. Equitable access to fisheries resources is of prime importance towards social sustainability [42].

Participation in the form of power-sharing is an important aspect in attaining social sustainability where the power and authority are decentralized and the management process ensures the involvement of both the stakeholders and the higher authorities. The absence of community engagement in the management process, exclusion of local traditional knowledge and emphasis on top-down resource management have been proven to be failed resource management processes as evidenced by the historic collapse of the North Atlantic cod fisheries [43]. Participation is therefore an essential component of management that promotes favorable social and ecological results [44], particularly when it comes to a common pool resource like fisheries [45].

Equity is another important aspect of fisheries management and conservation efforts and for ensuring long-term sustainability, it is essential to ensure social justice and fairness in the management process [11,46]. As social justice is a key indicator of good governance, good governance is a must to bring social sustainability [11]. According to Fabinyi et al. (2015), local fishers in the Philippines and Papua New Guinea were deeply troubled by feelings of inequity, which hindered governance and management [47]. It should come as no surprise that the FAO and the Too Big To Ignore (TBTI) working group have identified fairness and equality as fundamental management tenets for small-scale fisheries [48].

Gender equity is one aspect of equity that is commonly discussed in the literature on fisheries. In the fisheries sector, advancement toward gender equality is crucial for achieving successful and equitable development results [49]. Women have critical roles in the fisheries sector, but their contributions are usually unseen, frequently neglected and always unappreciated [50]. A little progress has been made so far in recognizing and implementing gender equality in the planning, management and development process of the fisheries sector [51]. Moreover, due to gender norms (societal and cultural expectations from women), their contribution to this sector is often recognized as unpaid, opportunistic, part-time and an extension of daily household responsibilities [52]. However, the exclusion of this huge portion of the viable community has negative consequences in the fisheries management process as well as in ensuring individual and community well-being [53]. In addition, shared prosperity can also be helpful for improving the socio-economic conditions of marginal fishers.

Various types of indicators may be used to assess how well a specific fishery satisfies the broad criteria of social sustainability [54]. The Social Assessment Handbook [55] specifies six different forms of data that can be acquired on fisheries They are the fishing history, social profiles, quality of life, social capital, values and beliefs of fishing groups and the broader community and community spatial analysis in respect to the fishery assets they utilize. A vital tool for comprehending the social dimensions of any given occurrence is quality of life. Three distinct types of data may be used to gauge one's quality of life. The first is financial indicators concerning people's income and purchasing power, which have been pretty extensively examined and documented worldwide throughout the decades. Other well-established social data, such as crime and health rates, make up the second category of indicators. Subjective data regarding how individuals feel about their lives and societies is the third sort of data. Subjective assessments of quality of life comprise general life gratification and job satisfaction [54].

The United Nations Sustainable Development Goals (SDGs) aim to safeguard the environment and enhance the opportunities and well-being of all people, everywhere [56]. The SDGs had 17 goals where Goal 1. No poverty, 2. Zero hunger, 5. Gender equality, 14. Life below water and 15. Life on land, directly or indirectly focuses on social sustainability. Each specific goal sets some specific targets to achieve the SDGs. For instance, Goal 1 (No poverty) lays out a number of objectives, including 1.1, 1.3, 1.4 and 1.5, which can be met by guaranteeing equitable access to fisheries resources and other essential services, like the ownership of natural resources, and by promoting the development of resilience against social, economic, and environmental calamities. Additionally, Goal 1 aims to eradicate poverty among those who depend on natural resources by increasing income through increasing fish production. Moreover, by attaining SDG's Target 2.3 by increasing fisheries productivity and providing equitable and safe entrance to resource users, knowledge of economic facilities, markets, and prospects for value addition, marine conservation measures may help achieve Goal 2 (Zero hunger) [56].

Moreover, in order to restore fish stock as quickly as possible – at least to a level where their biological characteristics allow for the maximum sustainable yield – marine fisheries management actions may help to achieve targets 14.b and 14.7 of Goal 14 (Life below water). They can also support small-scale fishermen and increase the monetary profits of the sustainable usage of marine assets by sustainable supervision of fisheries, aquaculture and tourism [56]. 10.13039/100014337Furthermore, fisheries management actions might assist in achieving Goal 15 (Life on land) by attaining SDG's 10.13039/100004791Target 15.6 by supporting the rationale allocation of profits rising through the exploitation of fisheries resources [56].

## 3 Methodology

### 3.1 Description of the study area

**Fig. 1:**
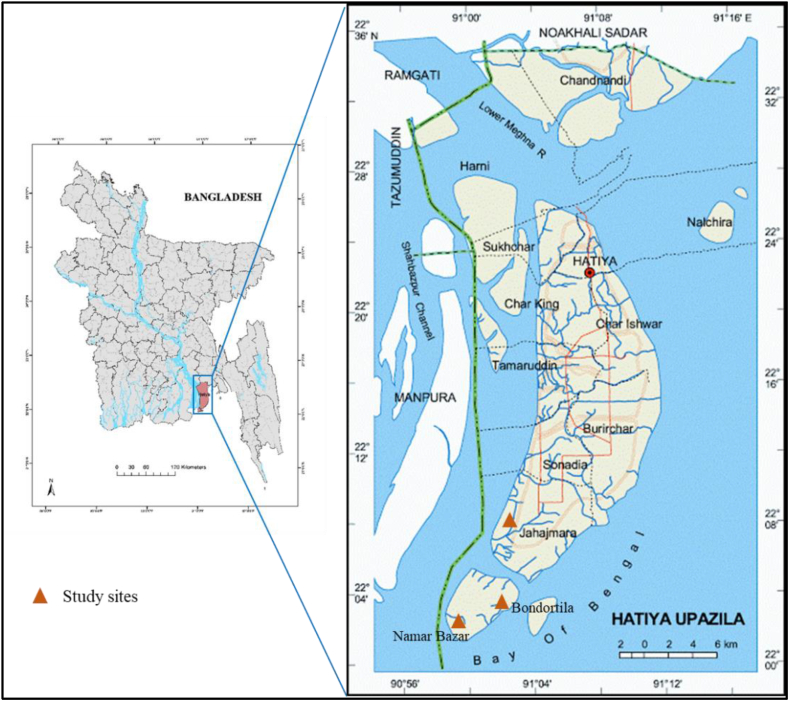
Study sites in Hatiya Upazila, Bangladesh.

The primary data for this study were collected from Hatiya upazila of Noakhali District (Fig. 1). Prior to final data collection, a scoping study was carried out to understand the study areas and livelihood activities of the local community members of Nijhum Dwip and Jahajmara unions at Hatiya upazila in August 2021. Based on the findings of the scoping study, three fishing communities namely Namar Bazar and Bandortila at Nijhum Dwip and Katakhali at Jahajmara (Fig. 1) were selected where the majority of the local people depend on fishery resources, particularly on marine fishing. Nijhum Dwip is a small, remote island in Bangladesh that is located in the Meghna Estuary, at the confluence of the Bay of Bengal (BoB). It is surrounded by the BoB on the south and west, Domar Char and the Meghna River on the east, and Hatiya Island on the north. The elevation of the island above sea level is about 2.2 m [57]. Nihum Dwip, covering an area of 163.45 km^2^, is located between latitudes 22°1′30″ N to 22°6′ N and longitudes 90°58′30″ E to 91°3′ E. More than 25,000 people lived in Nijhum Dwip in 2016, and the majority of them are engaged in hilsa fishing, fish drying, and agriculture-related activities [58]. On the other hand, Jahazmara is located in the Hatiya upazila in the district of Noakhali. The area of Jahazmara Union is 213.23 km^2^. Jahazmara Union is located in the south of the main island of Hatiya Upazila. Burirachar Union and Sonadia Union are in the north of this union, Hatiya Channel and Sakuchia North Union of Manpura Upazila of Bhola district in the west, Hatiya Channel and Nijhum Dwip Union in the south and the Bay of Bengal are located in the east. According to the 2011 census, Jahazmara Union has a population of 56,001 [58]. Alike Nijhum Dwip, the majority of the people are engaged in fishing, especially hilsa fishing, fish drying, small business, and agriculture-based functions. A comparative demographic characteristic of Jahajmara Union and Nihhum Dwip Union, Hatiya Upazila, Noakhali, Bangladesh is given in Table 1.

**Table 1:** Comparative demographic characteristics of Jahajmara Union and Nihhum Dwip Union, Hatiya Upazila, Noakhali, Bangladesh.

Variables	Jahajmara union	Nijhum Dwip union
Area (km^2^)	213.23^a^	163.45^a^
Population (number)	56,001^a^	25,000^a^
Family size (mean ± sd)	5.34 **±** 1.2^b^	5.5 ± 2.2^a^
Literacy rate (%)	34.4^a^	35^a^
Primary school (number)	29^a^	6^c^
Secondary school (number)	5^a^	1^c^
College (number)	1^a^	0
Madrasa (number)	3^a^	15^c^
Community clinic (number)	4^d^	3^c^
Access to medical facilities^b^	Moderate	Poor
Access to electricity^b^	Present	Absent
Women participation in fishery-related activities^b^	Low	Absent
Average monthly income (USD)	157^b^	113^e^
Major livelihood activities^b^	Fishing, fish drying, boat making, net mending.	Fishing, fish drying, boat making, net mending, tourism.

a BBS, 2011 [58].

b Semi-structured interviews and FGDs.

c BNP, 2023 [59].

d MoHFW [60].

e Islam et al., 2021 [61].

### 3.2 Data collection

This study used a mixed-method approach to gather both qualitative and quantitative data during August 2021 and February 2022. This study has obtained ethical clearance from the Ethical Review and Clearance Committee Involving Humans in Research, Faculty of Biological Sciences, University of Dhaka (Reference no.: 109-B/Biol. Scs. Date: August 10, 2021). Data collection tools are described in the following sections.

#### 3.2.1 Household questionnaire interview

In this study, household questionnaire interviews were carried out to obtain data on how the marine fishing ban has affected the dependent fishing community. A list of fishers was collected from the Hatiya Upazila Fisheries Office, which was used as the sampling frame for this investigation. The method of Yamane [62] was used to compute the sample size in this study as n = $\frac{N}{{1+N\;\left(e\right)}^{2}}$ = 162, where n represents the sample size, N is the population size (273) and e denotes the marginal error (0.05). In this study, simple random sampling technique was used to choose the respondents. Merely household heads were questioned for this study, and each interview continued for 45 min. Initially, a semi-structured questionnaire (in *English*) was designed and translated into the local language (in *Bangla*) which underwent a small-scale piloting to certify that the questions posed and the language employed were appropriate. Then the questionnaire was revised (if any uncertainty arose during pilot testing in wordings or understandings) for final data collection. Before conducting the surveys, informed consent (verbal) was taken from each participants to ensure their voluntary participation. However, taking written consent was not possible as the participants become suspicious and it lead to a loss of rapprt.

#### 3.2.2 Focus group discussions (FGDs)

In order to augment and validate the data obtained from questionnaire interviews from the fields, four FGDs were carried out in this study. A greater focus was placed on elucidating matters that appeared ambiguous during the household interviews. At each FGD, eight to ten homogenous respondents – indigenous fishermen who typically fish in the marine waters – were attended [63]. During household interviews, heads of fishing families who showed cooperation, knowledge, and enthusiasm were chosen for FGDs. Overall, the way the FGD sessions were conducted ensured that the questions were answered, the participants truly participated, and the focus and momentum were maintained. A checklist (in *Bengali*) was designed earlier for the FGD participants to discuss the pros and cons of marine fishing ban on the reliant fishing communities. To encourage each respondent to participate in the debate, a breakout session was held. Each session continued for around 2 h. Each FGD session was conducted in a place (e.g., the bank of the river where they moored their fishing vessels) that was comfortable for all of the fishers. Each EGD session was audio-recorded after obtaining participants' consent and transcribed later for data analysis.

#### 3.2.3 Key informant interviews (KIIs)

In this study, thirteen key informant interviews (KIIs) were carried out. The primary goal of conducting KIIs was to validate and have in-depth conversations about the significant topics that the respondents brought up in their FGDs and household interviews. For KIIs, persons (e.g., president of the local fishing association, upazila fisheries officer, chairman of the local union parishad and local fishers) who have sufficient knowledge about the fishing communities from both inside and outside the communities were selected. Like FGDs, a checklist (in *Bengali*) was also designed prior to the KIIs. Each session lasted for 40–45 min. KIIs were carried out by visiting the participants' sites of interest. Individualized KIIs were administered to guarantee the privacy of the answers. Alike FGDs, each KII session was also audio-recorded after obtaining the key informants' consent and transcribed later for data analysis.

### 3.3 Data analysis

In this study, descriptive statistics including mean, standard errors, frequency, percentages, Pearson's chi-square test, and so on were used to analyze quantitative data. Shortly after the interviews, the answers to the open-ended questions from the semi-structured interviews, FGDs, and KIIs were hand-written. The recorded data were transcribed in Bangla. The data's dependability was enhanced by cross-referencing the transcriptions with the recorded audio at least once to look for any discrepancies. The qualitative data were evaluated through content analysis technique and they were characterized into different themes and groups based on the grounded theory approach. The three steps of the analysis were to first prepare and arrange the data, then use coding and shortening the codes to reduce the data into themes, and lastly present the data using tables or through a discussion [64]. Data were shown in graphical and tabular formats.

## 4 Results

### 4.1 Demographic characteristics

The findings of this study demonstrate that 100 % of the participants are full-time fishers and all the fishing households are male-dominated i.e. male members are decision-makers of the households (Table 2). The household heads' mean age is 39.61 years. Around 90 % of the respondents of this study are illiterate with a mean years of education of 0.34 ± 1.17 years. Most of the fishers have been fishing for over a decade with an average fishing experience of approximately 18.53 **±** 8.34 years. But only 38 % of the fishers hold government-provided fisher's identity and the rest of the fishers have none. Fishers use different kinds of fishing gear and craft to catch fish including gill nets (*chandi jal*) (85 %), purse nets (*behundi jal*) (56 %) and seine nets (*sutar jal)* (26 %) together with cast nets and other types of nets. Around 97 % of the fishers use non-motorized boats during fishing. Around 83 % of them work on other fisher's boats whereas only 15.3 % have their boat or shared ownership of the boat. The monthly average income of the fisher's households from fishing and other activities is around USD 157.41 ± 133.43 whereas the average monthly expenditure is USD 126.74 ± 77.71.

**Table 2:** Demographics of the studied households at Hatiya, Noakhali, Bangladesh (n = 162).

Categories			Percentage	Mean ± SD
Fishers type	Full time		100	–
Gender	Male		100	–
Age (years)	<18		0	39.61 <b±></b> 11.58
	18–29		17	
	30–39		38	
	40–49		23	
	50–59		14	
	60 ≤		8	
Years of fishing	–		–	18.53 <b±></b> 8.34
Year of Education	–		–	0.34 ± 1.17
Number of household family members	≤4		23.60	5.34 <b±></b> 1.18
	5–7		75.20	
	8–10		1.30	
	11 ≤		0	
Resources related to fisheries	Fishing boat	Mechanized	3	–
		Non-mechanized	97	
	Ownership of the boat	Owner	15.30	
		Shared	1.30	
		Worker	83.40	

Household's average expenditure (USD/month)^a^		126.74 ± 77.71
Household's average income from fishing (USD/month)	Outside ban period	137.13 ± 103.46
	During ban period	0
Household's total average income from other activities (USD/month)	Outside ban period	157.41 ± 133.43
	During ban period	20.28

a 1 USD = 87.62 BDT (date: May 30, 2022).

The relationship amid diverse categorical answers of the fishery-reliant communities, for instance, household head's age and fishing experiences, number of household family members and age of the household heads, monthly expenditure of the household and monthly income of the households from fishing, etc. are significant (Spearman's correlation matrix) (Table 3).

**Table 3:** Spearman's correlation among different categorical responses collected from the fisher's households in Hatiya, Noakhali, Bangladesh.

Variables	Fishing experiences (year)	Household head's age (year)	Year of education	Household member (no.)	Household expenditure (USD/month)	Household's fishing income (USD/month)
Fishing experiences (year)	1.000	0.523^a^	−0.134	0.178^b^	0.001	0.013
Household head's age (year)	0.523^a^	1.000	−0.172^b^	0.315^a^	0.104	0.090
Year of education	−0.134	−0.172^b^	1.000	0.021	0.008	−0.066
Household member (no.)	0.178^b^	0.315^a^	0.021	1.000	0.170^b^	0.028
Household expenditure (USD/month)	0.001	0.104	0.008	0.170^b^	1.000	0.826^a^
Household's fishing income (USD/month)	0.013	0.090	−0.066	0.028	0.826^a^	1.000

a Significant correlation at 0.01 level (2-tailed).

b Significant correlation at 0.05 level (2-tailed).

### 4.2 Impacts of marine fishing ban on fish catch

Fishers' perception of the changes in fish catch due to the implementation of the 65-day marine fishing ban followed a steady pattern. In this study, more than 50 % of fishers claimed that the overall fish catch was comparatively increased after the implementation of the marine fishing ban. The FGD participants described that the amount of fish they caught before the implementation of the fishing ban had increased later when the fishing ban started (Table 4). Overall both big and small-sized fish catches have increased after the implementation of the fishing ban. For example, the catch of large-sized hilsa (>1.2 kg and 0.5–1.2 kg) was also greater after the implementation of the fishing ban. However, according to 45 % of fishers, the catch of juvenile hilsa (size <25 cm) was higher before the implementation of the fishing ban which might have happened because of indiscriminate fishing of hilsa when there was no fishing ban. According to FGD participants, the number of big and medium-sized hilsa catches increased after the implementation of the fishing ban because of fishing closure in the marine water.

**Table 4:** Fishers’ perception (%) on fish catch before and after 65 days marine fishing ban implementation in the Bay of Bengal.

Indicators	65 days fishing ban implementation	Fishers' perception (%)				
		Very high	High	No change	Low	Very low
The overall catch of fish	Before	7	32	4	57	0
	After	7	52	5	35	1
Big-sized fish catch	Before	4	40	6	50	0
	After	3	47	7	40	3
Small-sized fish catch	Before	1	27	28	44	1
	After	12	35	29	22	2
>1.2 kg size hilsa catch	Before	9	39	6	46	0
	After	1	47	5	45	2
0.5–1.2 kg size hilsa catch	Before	3	27	19	50	1
	After	10	46	24	20	
Juvenile hilsa catch	Before	13	45	10	23	9
	After	7	23	10	33	27

### 4.3 Impacts of marine fishing ban on daily basic needs

In this study, it was reported that the 65-day marine fishing ban negatively affected the fisher's basic daily needs, especially their revenue generation from fishing was decreased throughout the ban (Table 2). The food consumption of around 33 % of fishers' households has drastically reduced throughout the ban compared to the fishing period (Table 5). The consumption of fish is relatively high in fishing communities as they consume less-valued fish species and sell high-valued species. The weekly fish consumption of the fisher's households has decreased from 7 kg to 4 kg during the ban (Table 6) as they cannot afford to purchase fish from the market as claimed by the FGDs participants.

**Table 5:** Fishers’ perception (%) on the impact of 65 days marine fishing ban on their daily basic needs.

Indicators	During/outside the ban period	Fishers' perception (%)				
		Very good	Good	Moderate	Bad	Very bad
Daily food consumption	Outside ban period	17	50	30	3	0
	During ban period	1	16	49	33	1
Household relationships	Outside ban period	5	55	31	8	1
	During ban period	1	21	30	41	7
Healthcare facilities	Outside ban period	3	36	48	13	0
	During ban period	1	13	50	29	7
Children's education	Outside ban period	3	27	47	20	3
	During ban period	0	6	51	29	14

**Table 6:** Changes in weekly fish consumption (kg/week) of the households due to the 65-day fishing ban period (n = 162).

Time period	Range of fish consumed (kg per week)	Average ± SDd (kg per week)
During 65 days fishing ban period	0–16	4 ± 3.44
Outside the 65 days fishing ban period	1–20	7 ± 4.24

Around 41 % of fishers reported that their household relationships had been worsening during the ban as they had no income at that time. The FGD participants reported that they had nothing to do outside during the fishing ban, so they stayed at home most of the time. As there is no source of income during the fishing ban, the fishers' mood becomes irritable at that time. This often leads to quarrels with their wives over trivial matters. One of the key informants also reported that household relationships weaken because of the economic hardships of the households. However, the context is different when there has been no fishing ban over the years. Around 55 % of fishers reported that their household relationships were good when there was no fishing ban compared to the relationships during the fishing ban. The study also found that the healthcare facilities, children's education, and fisher's opportunities for supplementary work had been adversely affected due to the ban period (Table 5).

### 4.4 Impact of marine fishing ban on women and children

**Fig. 2:**
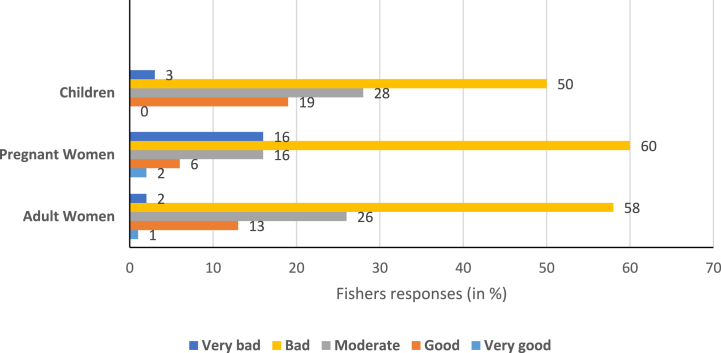
Impacts of the fishing ban on children, pregnant women and adult women based on fisher's perception (in %) in Hatiya, Bangladesh.

The fishing ban has an extreme effect on the children and women of the studied fishing communities. According to 50 %, 60 %, and 58 % of the respondents, the impacts of the fishing ban are bad for children, pregnant women and adult women (respectively) of the studied households (Fig. 2). Particularly fishing ban created negative impacts on the financial circumstances of the reliant communities which ultimately affected the children and women of the households. In this study, FGD participants also revealed that reduction of nutritious food intake negatively affects pregnant women, adult women, children and the elderly members of the households. This study has found that household food consumption has reduced from three meals to two or one meal and in that case, female household members suffered most as particularly they eat less. One of the key informants also reported that the healthcare facilities of women of the fisher households were harshly affected during the ban period and the pregnant women and children seriously suffered from protein deficiency during this time.

### 4.5 Impact of marine fishing ban on Fisher's finance

**Fig. 3:**
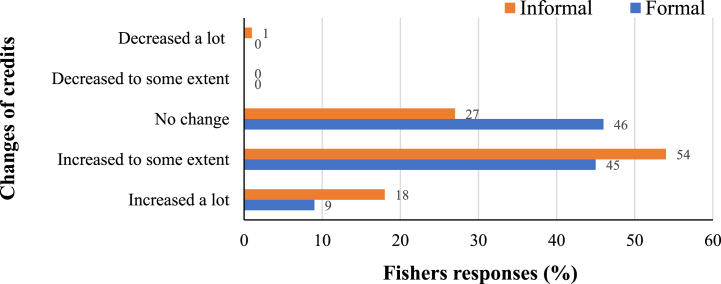
Changes of credits during the 65-day fishing ban on the fishery-dependent communities of Hatiya, Bangladesh.

This study found that the average monthly fishing income of the fisher's household is USD 137.13 ± 103.46 outside the fishing ban period. However, they cannot earn any revenue during the ban as they cannot go fishing during this time. Some fishers can earn some money from alternative jobs which is not enough to bear their family expenses (see section 4.6 for more details). Approximately 71 % of fishers have to take loans from various sources, for example, local money lenders (*Mohajon* or Aratdar). The fishers' propensity to take both formal and informal loans increases during the ban period as reported by 45 % and 54 % of respondents, which is mainly to maintain household expenses (Fig. 3).

The FGD participants also claimed that a substantial number of fishers have no alternative income options and thereby are compelled to take the loan. The FGDs also revealed that sometimes children are also engaged in income-generating activities which ultimately affects their education.

### 4.6 Impact of marine fishing ban on alternative-income generating activities

This study has found that fishing is the key income-generating option for almost all of the studied households (see Table 2). There is little scope for alternative livelihood opportunities for them. Only 13 % of fishers have alternative income-generating options such as daily wage earning, day laboring in agricultural fields, agriculture, rearing cattle, small business, carpentry, driving, etc. Around 32 % of fishers reported that the opportunity for alternative livelihood options became worse during the ban (Table 7) as fishers became jobless at that time. In addition, many fishers have no skills for other income-generating activities except fishing.

**Table 7:** Fishers’ perception (%) on the alternative livelihood options during and outside the 65-day marine fishing ban.

Indicators	Timeframe	Fishers' perception (%)				
		Very good	Good	Moderate	Bad	Very bad
Alternative livelihood options	Outside ban period	13	15	17	48	8
	During ban period	0	2	14	52	32

### 4.7 Compensation scheme and marine fishing ban

**Fig. 4:**
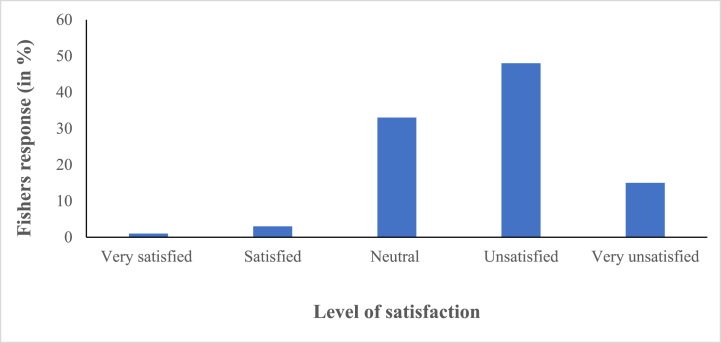
Level of satisfaction for the compensation during the 65-day marine fishing ban in Bangladesh.

In this study, around 50 % of the fishers were unsatisfied with the compensation given during the 65-day fishing ban period (Fig. 4). Mismanagement in the dissemination process has been reported by the fishers. Only the fishers with ID cards get the compensation provided by the local government members and they even favor their family, friends and relatives while distributing the compensation. The FGD participants reported that 40 kg of rice for one month is an inadequate amount compared to their household needs. Also, they cannot meet other household expenses like education and health expenses. This study has reported that fishers are not concerned with the 65-day fishing ban implementation process. Therefore they showed dissatisfaction regarding the ban implementation.

## 5 Discussion

### 5.1 Implications of 65 days marine fishing bans

As per this study, 65-days marine fishing ban has positive impacts on overall fish production despite the significant negative impacts on the reliant fishing societies. Similar findings were also reported in some other studies such as [25,65,66]. Islam et al. (2018) reported an optimistic trend of fish catch from a majority of fishers after the implementation of conservation action like the establishment of hilsa sanctuary in the Meghna River [65]. Hossain et al. (2017) also reported that fish sanctuary establishment has a positive impact on fish biodiversity and production in the coastal regions of Bangladesh [67]. One study in India showed that 60 days marine fishing ban assisted in the restoration of habitat and rebuilding of stock through recruitment [14] which is in line with the findings of the present study.

This study has shown that the 65-day marine fishing ban impacts fisher household's food consumption particularly fish consumption drastically reduced throughout the ban period. Islam et al. (2016a) also reported that many fishers and their household members are unable to consume any fish during the ban [25]. As fish is the key source of protein in the fishers' community, during the ban the fishers do not get the opportunity to eat fish at all which does not support the social sustainability of the reliant on societies. Islam et al. (2016b) reported that not having proper and enough nutritious food over a substantial period may have a severe impact on the health of children and pregnant women [24]. As the women and children of the fishing communities suffered the most which is not an indication of social sustainability [2].

This study has found that the average monthly income of the fishers from fishing was USD 137.13 (12,015 tk) excluding the ban period which is 37 % (2.7 folds) lower than the overall national households mean monthly income of USD 370 (32,422 tk) of Bangladesh in 2022 [68]. With this income, the fishers have faced great difficulties in managing their family expenses. The market price of some basic food items is - rice (coarse) - 55 tk/kg, soybean oil (loose) - 154 tk/liter, lentils (local) - 95 tk/kg, potatoes - 35 tk/kg, onions - 64 tk/kg, chilies (green) - 120 tk/kg, salts - 38 tk/kg, eggs - 50 tk/4 pieces, fish (tilapia/pangas) - 200 tk/kg, and chicken (broiler) - 200 tk/kg, etc. (based on local market data, October 24, 2023). Given the current market price of the daily essential commodities, a fisher family of four has to spend 60–70 % or more of their income per month only for food consumption [69]. As a result, it becomes hard for the fishers to manage their household expenditures with the meager income from fishing. In addition, the fishers became jobless during the 65-day fishing ban and as a result, they had no income from fishing which severely affected their daily basic needs. Eriksson et al. (2019) [70] showed that the fishing ban in Indonesia had also adversely affected the fisher's daily basic needs as their income had dramatically reduced during the ban which strongly supports the results of this study. Overall, the daily basic needs of the fishery-reliant societies are affected by the marine fishing ban.

To cope with the adverse situation during the fishing ban, most fishers took informal loans from the local money lenders (*mohajons* or *aratdars*) and only a few took loans from NGOs. This finding is in line with Siam et al. (2020) who reported that 56 % of the fishers of the Nijhum Dwip area borrowed money (informal) from moneylenders and 44 % from non-government organizations (NGOs) [71]. Besides taking formal or informal loans, fishers often engaged their school-going children in non-fishery-related economic activities to support their families during the hard times which ultimately affected children's education. One study reported that the school dropout rate was higher for boys (63 %) in the Nijhum Dwip area compared to girls (37 %) as they are often involved in household earnings [71]. As a result, fishers and their household's social sustainability are neglected due to environmental or ecological sustainability. However, the social sustainability of the fishery-reliant peoples is a critical requirement. Security of the daily basic needs of fishers' families during the fishing ban must be ensured in order to achieve social sustainability.

This study has found that the lack of alternative job skills hinders the socio-economic condition of the fishery dependent stakeholders. Rahman et al. (2012) also reported some constraints that affected the local community members' livelihood activities in the Hatiya area [72]. The main constraints were the burden of loans, market control by local money lenders, inadequate market facilities, pirates in the fishing areas, low wages, and limited land access for agriculture and inadequate technological support in both the boat and landing center. The lack of gender-friendly income-generating activities is also another main constraint in that area [73]. Eriksson et al. (2019) found that around 46 % of the fishermen of Indonesian fishing communities had no access to alternate income-generating activities due to the lack of access to financial assistance to start new businesses, which are not the general features of a sustainable society [70].

Some initiatives have been taken by the government, for example, vulnerable group feeding (VGF) activities (40 kg of rice/registered fisherman/month) and/or alternative income-generating options throughout the fishing ban period to support the fishers [74]. However, around 97 % of fishers reported that, in 2021, they did not get the incentives at all. The key informants explained that the inadequate allocation of incentives from the government is the main reason for this outcome. Islam et al. (2023) reported that more than 50 % of the local fishers are very unsatisfied with the compensation scheme in the Hizla-Mehendiganj, Barishal. Moreover, they do not get their compensation on time [13]. However, compensation should be provided on time as per the government's rules i.e., before and during the implementation of the fishing ban [75]. Islam et al. (2018) also reported corruption and nepotism in the enlistment process of legitimate fishers [65]. The household questionnaire survey respondents and FGD participants reported that the fishers do not support the ban and are largely dissatisfied with its implementation which strongly supports the findings of some studies [25,65,76]. The above-discussed issues are not aligned with the characteristics of a sustainable society as social sustainability ensures the part-time and full-time jobs for the individual of a community, meet the basic needs and requirements for survival and growth.

### 5.2 Social sustainability in the marine fishing community

To facilitate fish reproduction, the Fisheries and Livestock Ministry had previously published a notice in the gazette on May 20, 2015, banning fishing in the bay from May 20 to July 23, annually. Encouraging everyone to take action to protect marine resources, as fish productivity would increase if the prohibition on fishing during the restricted period could be effectively implemented. However, recognizing the possible consequences of the fishing restrictions, several hundred fishers protested the 65-day ban on fishing in the Bay of Bengal by setting up a rally at different places over the country with banners, placards, and festoons. Subsequently, a writ petition contesting the ban's validity was submitted to the High Court. On May 15, 2017, the government order was affirmed by the High Court. To protect fish and crustacean species that spawn, the Bay of Bengal saw the imposition of a ban on all forms of fishing in 2019. The ban initially applied only to industrial trawlers. The fishing prohibition is helping Bangladesh produce more fish, but it is also causing economic loss and threatening the social sustainability of the fishery-dependent communities along Bangladesh's coast [13,65,77].

Around the world, numerous conservation initiatives such as marine fishing bans, marine protected areas, etc. have been undertaken to address overexploitation and advance fisheries sustainability. Like 65 days of marine fishing ban in Bangladesh, 60 days of marine fishing ban in Indian coastal waters [14] and three months long marine fishing ban in the Visayan Sea and adjoining waters of Philippines [28] are present. The adverse impacts of fishing bans on the livelihoods of the fishers that have been reported in this study are aligned with the results of Infantina et al. (2020) [14] and Napata et al. (2020) [28]. In addition, marine protected areas or marine reserves where fishing is temporarily or permanently closed have created negative impacts on the social sustainability of fishery-dependent communities [70,78,79].

According to the City of Vancouver (2005), the capacity to conserve and build on its resources along with the capacity to discourse future difficulties creates a sustainable society [3]. However, in the Hatiya area, the study has seen different situations during the marine fishing ban. Fishers as well as their household members particularly women have suffered a lot during the ban. Their daily basic needs and fish consumption are reduced drastically at that time which is not a feature of a socially sustainable community. Fishers also became indebted during the ban. An important component of social sustainability is safety and security which is missing in Hatiya. Fishers do not have any security for employment and income during the ban. They do not have any safety for daily basic needs. Another issue is the inadequate compensation scheme during the fishing ban. In addition, equitable distribution of compensation schemes is missing in that area. During the ban, there are few alternative income-generating options for males which are not enough. One of the key informants also reported that inadequate or lack of women-friendly income-generating activities (IGAs), women household members cannot earn a little. As a result, women household members cannot support their male counterparts a little to bear the household expenses. Though gender equality is a widely decided principle and has been embraced extensively, its implementation in different sectors varies differently [51]. The above-discussed issues indicate that the fishing communities of the Hatiya area are not socially sustainable.

### 5.3 Way forward toward social sustainability

Though the 65-day marine fishing ban has shown an affirmative impact on fish production, the socio-economic conditions of the fishers and their household members have been adversely affected leading to poor social sustainability. The study has focused on some important issues that have the potential to bring social sustainability to Bangladesh's fisheries management and contribute to partly meeting the targets of Sustainable Development Goals 1, 2, 14 and 15. These are outlined below:

a. **Enough compensation scheme during the fishing ban:** Ensuring equitable, sufficient and transparent distribution of the compensation schemes is important for sustainable management of the marine fishing ban. The government should make sure that all ban-affected fishers easily get the allotted compensation scheme. The distribution process should be fair and transparent and adequate to sustain the basic needs of the fisher's households.
b. **Fulfilling protein demand during the ban:** Providing only rice as compensation is not adequate to meet the dietary demand of the fisher's households. During the fishing ban, fishers cannot catch fish and thus cannot consume fish that is why the compensation scheme should also include protein sources like fish, meat, etc. to meet their protein needs.
c. **Creation of part-time and full-time alternative job opportunities:** Most of the fishers in Hatiya are completely reliant on fishing. Due to limited alternative livelihood opportunities, the long-term ban affects their income. The GOs and NGOs functioning in this area should provide them with different alternative livelihood options based on their needs like small businesses, training on handicrafts, sewing machine operation, net mending, cage culture, cow fattening, livestock rearing, plant nurseries, homestead gardening, etc. for both male and female members of the fisher's household as ensuring equitable income is a prerequisite for social sustainability.
d. **Capacity development:** This study has found that many fishers have no skills for other income-generating activities except fishing. So, the government can take the initiative to develop fishers' skills. For this, the government should allocate a budget for the capacity development of the fishers and their household members for long-term solutions to accomplish social sustainability. The budget might be used to train the fishers to develop their skills for alternative job activities. Kaewnuratchadasorn et al. (2020) also suggested that in Southeast Asian regions, creating alternative livelihoods and alternative marketing systems can enhance the capacity of the fishers [80].
e. **Financial support for fishers' households during the ban:** The government should consider providing financial assistance to fishers during the fishing ban to promote social sustainability in fisheries management, as all of the fishers stated that they have to buy other daily goods like groceries and children's education and healthcare. Policymakers can also consider including other daily necessities in the compensation package, for example, oil, pulses, salts, and so on, which are important for households.
f. **Establishment of a fishery bank:** This study has found that around 71 % of fishers became indebted during the fishing ban because of inadequate income. They don't have access to bank loans due to collateral deficiency and in most cases take loans from NGOs (micro-credits) and local money lenders with high interest rates and unfavorable terms and conditions. Pomeroy et al. (2020) reported geographic barriers and lack of formal identification also influenced fishers to take informal loans [81]. Thus, a dedicated fishery bank needs to be established for fishers only. Among the 60 scheduled banks registered in Bangladesh, none is devoted to fisheries. The establishment of a fishery bank is thus imperative for the fisheries sector for social sustainability.
g. **Increasing awareness and participation in fisheries management:** The study has found that the fishers have little knowledge about the implementation of conservation activities in their region. So, appropriate consciousness sessions should be organized to notify the fishers about the conservation implementation and management process and their significance in both the ecosystem and their life and livelihoods. The Department of Fisheries can contribute significantly to this initiative to raise awareness. In addition, the participation of the resource users is very crucial for bringing social sustainability in fisheries management.
h **Insurance coverage**: It is also important to bring the fishery stakeholders under insurance coverage since they have no scope to earn revenue during the ban period. For example, FAO (2020) recommended providing payroll and unemployment support for crew members and small fish farmers during the pandemic situation due to COVID-19 as they had no/very little income from fisheries [82]. So, insurance coverage due to the impacts of the fishing ban might bring social sustainability for the poor fishers of Bangladesh.

## 6 Conclusion

Fisheries management and conservation strategies such as the 65-day marine fishing ban are vital initiatives to develop hilsa fisheries as well as fish biodiversity in marine habitats. At the same time, the social sustainability issues of the fishery-dependent communities need to be considered. The socio-economic impacts of a marine fishing ban in Bangladesh have been assessed in this study. The marine fishing ban affected the fishers and their households' basic needs, food consumption, health facilities, children's education, household relations, and opportunities for alternative work during the ban despite positive impacts on fish production. This study has shown that female household members were the worst sufferers throughout the fishing ban. Fishers are often indebted due to the lack of earnings from fishing during the ban. Moreover, the compensation provided by the government during the ban is not enough. Considering the socio-economic impact of the marine fisheries management in the studied area, this study has shown some way forward for bringing social sustainability in marine fisheries management like proper and enough allocation as well as fair distribution of compensation scheme, including groceries in the compensation scheme, alternative income-generating options, etc. The incorporation of social sustainability criteria and indicators, such as food provision, in marine fisheries conservation actions may enhance fisheries management and future policies. The conclusions of this study might assist policymakers to better manage conservation actions by involving the fishers and considering their socio-economic hurdles during the ban period to ensure sustainable fisheries management.

This study is particularly focused on Hatiya upazila-based fishing communities that mostly depend on the marine fisheries sector of Bangladesh. Alike Hatiya upazila fishing communities, there are many fishing communities in different coastal upazilas of Bangladesh, however, we did not collect comprehensive data from all of the fishing communities in this study. As a result, there might be a weakness in making statistical inferences (generalizations) of this study due to the sample size, however, the findings of this study may raise the questions of social sustainability in the national as well as international platforms. The outcomes of such a study can also help in understanding the social sustainability issues in other parts of Bangladesh with similar ecosystem conservation, employment and livelihood circumstances. Moreover, this study used a mixed-method approach (both qualitative and quantitative methods) for assessing the social sustainability of the local fishers, but social sustainability indicators based mixed-method approach might provide a more robust outcome of the impacts of the marine fishing ban. Thus, this study suggests comprehensive insights into the social impacts of conservation actions such as marine fishing bans which can be further explored with greater sample sizes and in other places. The robustness of the results is enhanced by the use of mixed methods and multi-staged sampling techniques. This type of cross-national comparison research may also enable nations to share knowledge.

## Data availability statement

Data will be made available upon request. Please email the corresponding author.

## CRediT authorship contribution statement

**Md Monirul Islam:** Writing – review & editing, Writing – original draft, Validation, Supervision, Project administration, Methodology, Funding acquisition, Conceptualization. **Makidul Islam Khan:** Writing – review & editing, Writing – original draft, Visualization, Methodology, Investigation, Formal analysis, Data curation. **Gouri Mondal:** Writing – review & editing, Methodology, Investigation, Data curation. **Most Nilufa Yeasmin:** Writing – review & editing, Writing – original draft, Visualization, Formal analysis, Data curation. **Aparna Barman:** Writing – review & editing, Visualization, Methodology.

## Declaration of competing interest

The authors declare that they have no known competing financial interests or personal relationships that could have appeared to influence the work reported in this paper.
