# Corrigendum to “Social sustainability in Bangladesh marine fisheries management: A case from Hatiya fishing community” [Heliyon Volume 10, Issue 14, July 2024, Article e34124]

**DOI:** 10.1016/j.heliyon.2025.e43450

**Published:** 2025-05-30

**Authors:** Md Monirul Islam, Makidul Islam Khan, Gouri Mondal, Most Nilufa Yeasmin, Aparna Barman

**Affiliations:** a Department of Fisheries, University of Dhaka, Dhaka, 1000, Bangladesh; b Department of Fisheries and Marine Bioscience, Bangabandhu Sheikh Mujibur Rahman Science and Technology University, Gopalganj, 8100, Bangladesh

In this article, the explanation for the absence of written informed consent requires clarification to align with ethical reporting standards. The original statement omitted the critical reason for not obtaining written consent (participants' low literacy levels). This omission created ambiguity about the ethical justification for using verbal consent. The corrigendum involves explicitly stating that written consent was not feasible due to participants’ limited literacy, necessitating verbal consent to ensure comprehension while maintaining transparency about the method used.

Original version of the informed consent statement.

Before conducting the surveys, informed consent (verbal) was taken from each participant to ensure their voluntary participation. However, taking written consent was not possible as the participants became suspicious, and it led to a loss of rapport.

The authors clarify that written consent was not obtained due to participants’ low literacy levels, which made written documentation impractical and potentially exclusionary. The revised explanation retains the acknowledgment of verbal consent while explicitly linking the choice to literacy barriers.

The correct version of the informed consent statement should be as below.

Before conducting the surveys, informed consent (verbal) was obtained from each participant to ensure their voluntary participation. Written consent was not feasible due to participants’ limited literacy levels, so verbal consent was prioritized to ensure comprehension while minimizing participant discomfort.

The authors apologize for the errors.

## Declaration of competing interest

The authors declare that they have no known competing financial interests or personal relationships that could have appeared to influence the work reported in this paper.

